# Pyruvate dehydrogenase kinase as a novel therapeutic target in oncology

**DOI:** 10.3389/fonc.2013.00038

**Published:** 2013-03-07

**Authors:** Gopinath Sutendra, Evangelos D. Michelakis

**Affiliations:** Department of Medicine, University of AlbertaEdmonton, AB, Canada

**Keywords:** mitochondria, tumor metabolism, dichloroacetate, pyruvate dehydrogenase kinase, hypoxia-inducible factor 1α, apoptosis resistance

## Abstract

Current drug development in oncology is non-selective as it typically focuses on pathways essential for the survival of all dividing cells. The unique metabolic profile of cancer, which is characterized by increased glycolysis and suppressed mitochondrial glucose oxidation (GO) provides cancer cells with a proliferative advantage, conducive with apoptosis resistance and even increased angiogenesis. Recent evidence suggests that targeting the cancer-specific metabolic and mitochondrial remodeling may offer selectivity in cancer treatment. Pyruvate dehydrogenase kinase (PDK) is a mitochondrial enzyme that is activated in a variety of cancers and results in the selective inhibition of pyruvate dehydrogenase, a complex of enzymes that converts cytosolic pyruvate to mitochondrial acetyl-CoA, the substrate for the Krebs’ cycle. Inhibition of PDK with either small interfering RNAs or the orphan drug dichloroacetate (DCA) shifts the metabolism of cancer cells from glycolysis to GO and reverses the suppression of mitochondria-dependent apoptosis. In addition, this therapeutic strategy increases the production of diffusible Krebs’ cycle intermediates and mitochondria-derived reactive oxygen species, activating p53 or inhibiting pro-proliferative and pro-angiogenic transcription factors like nuclear factor of activated T cells and hypoxia-inducible factor 1α. These effects result in decreased tumor growth and angiogenesis in a variety of cancers with high selectivity. In a small but mechanistic clinical trial in patients with glioblastoma, a highly aggressive and vascular form of brain cancer, DCA decreased tumor angiogenesis and tumor growth, suggesting that metabolic-targeting therapies can be translated directly to patients. More recently, the M2 isoform of pyruvate kinase (PKM2), which is highly expressed in cancer, is associated with suppressed mitochondrial function. Similar to DCA, activation of PKM2 in many cancers results in increased mitochondrial function and decreased tumor growth. Therefore, reversing the mitochondrial suppression with metabolic-modulating drugs, like PDK inhibitors or PKM2 activators holds promise in the rapidly expanding field of metabolic oncology.

## INTRODUCTION

Traditional drug development in oncology has focused on pathways that are essential for the survival of all cells. The major limitation of this approach is the selectivity of treatment to cancer cells. Alternatively, selectivity to cancer cells can be achieved by targeting *non-essential* pathways that are only critical for the survival of cancer cells, but this approach has limited efficacy. Overall, it is difficult to target both selective and essential pathways in current oncology, although there are exceptions. For example, chronic myelogenous leukemia (CML) cells dependence on BCR-ABL tyrosine kinase is induced by a chromosomal translocation only in the malignant cells (Rowley, 1973), making Gleevec a selective and effective treatment for CML (Kamb et al., 2007). Similarly, herceptin, an antibody that inhibits human epidermal growth factor receptor 2 (HER2) on HER2-positive breast cancers (Eisenhauer, 2001; Slamon et al., 2001) is also selective and effective, but like Gleevec this is an exception in oncology. In addition, most cancers are heterogeneous in nature and can adapt when non essential factors are targeted with non-essential therapy. For example, in glioblastoma multiform (GBM), even within the same tumor, one cell may have a different molecular abnormality than its neighbor cells, making the development of effective therapies very difficult, keeping the survival of these patients impressively low (Wen and Kesari, 2008).

In order to address this heterogeneity in oncology, integrative pathways that are also essential for the survival of cancer, but not normal cells, need to be targeted. Targeting such a pathway “distally” may address the fact that several “proximal” signals (for example several different oncogenes) maybe activated in any given cancer. The unique metabolism of most solid tumors integrates many molecular and genetic proximal signals, which all result in a switch in metabolism from mitochondria-based glucose oxidation (GO) to cytoplasm-based glycolysis even under normoxia, also known as the Warburg effect (Warburg, 1956; Michelakis et al., 2008; Vander Heiden et al., 2009; Dromparis et al., 2010). This metabolic profile may offer selectivity since it clearly separates cancer from non-cancerous tissues. This is evident by the very high uptake of glucose measured by positron-emission tomography (PET) in cancer, compared to the neighboring non-cancer tissues, making PET one of the most sensitive tools to diagnose cancer. At the same time, it is now clear that this metabolic switch offers a survival advantage to cancer cells and a resistance to apoptosis, perhaps forming an essential pathway for cancer, but not normal cells. Therefore, by reversing this mitochondrial remodeling, it is possible to “unlock” these cells from a state of apoptosis resistance, selectively inducing cancer cell death. A critical mitochondrial enzyme and a gatekeeper of GO is pyruvate dehydrogenase (PDH), which exists in a complex with its inhibitor, PDH kinase (PDK). There is now evidence that several oncogenes or transcription factors critical for cancer progression, like loss of p53 (Contractor and Harris, 2012) or activation of hypoxia-inducible factor 1α (HIF1α; Kim et al., 2006), can induce PDK expression and thus inhibit PDH and GO. Here we discuss the pre-clinical and clinical evidence that promoting GO with PDK inhibitors or similar approaches may be a novel approach in metabolic oncology.

## A METABOLIC SHIFT TOWARD GLYCOLYSIS OFFERS A PROLIFERATIVE ADVANTAGE TO CANCER CELLS

Most cancer cells use glycolysis as the primary energy source, an event that occurs early during the evolutionary progression of cancer. Gatenby and Gillies (2004) proposed that since early carcinogenesis often occurs in a hypoxic microenvironment, these cells must rely on anaerobic glycolysis as a primary energy source. This adaptation is initiated, in part, by activation of HIF, a transcription factor activated upon hypoxia or “hypoxia-mimicking” states. Once activated, HIF can regulate the expression of many glycolytic enzymes, glucose transporters, and mitochondrial enzymes (Semenza, 2012). One such enzyme important in regulating mitochondrial activity and induced by HIF is PDK (Kim et al., 2006). PDK is a gate-keeping mitochondrial enzyme that regulates the flux of carbohydrates via pyruvate from the cytoplasm into the mitochondria, where GO occurs. By inhibiting its target enzyme PDH, PDK prevents the coupling of glycolysis to GO. Therefore glycolysis is completed in the cytoplasm, where pyruvate is metabolized further without being oxidized in the mitochondria. This metabolic shift is a fundamental response in most tissues under hypoxia.

However, most cancer cells maintain the same metabolic phenotype when studied under normal, normoxic conditions. For example, the HIF1α-driven induction of PDK that occurs in the hypoxic microenvironment in early stages of carcinogenesis is maintained when tumor angiogenesis initiated by HIF1α increases oxygen supply to the growing tumor. There is now evidence that activation of several oncogenes can actually result in a similar suppression of mitochondrial function and inhibition of GO, independent of hypoxia. In other words, non-hypoxic signaling of several diverse oncogenes can mimic the effects of hypoxia and HIF1α and sustain the same metabolic phenotype, continuing to offer a growth advantage and apoptosis inhibition in the growing tumor.

For example, p53 inhibits the expression of the glycolytic enzyme phosphoglycerate mutase (Kondoh et al., 2005), while increasing the expression of the glycolytic inhibitor Tp53-induced glycolysis and apoptosis regulator (TIGAR; Bensaad et al., 2006). Additionally, p53 can regulate mitochondrial activity by increasing the expression of the mitochondrial cytochrome c oxidase subunits (Okamura et al., 1999; Matoba et al., 2006) and the mitochondrial DNA repair protein p52R2 (Bourdon et al., 2007). In other words, loss of p53, one of the most common molecular abnormalities in cancer, contributes to the glycolytic environment in cancer promoting glycolysis and inhibiting mitochondrial GO and function (Burns and Richter, 2008; Vousden and Ryan, 2009). Recent data also show that loss of p53 directly induces the expression of PDK and also enhances the transcriptional activity and stability of HIF1α ( Ravi et al., 2000; Kaluzova et al., 2004; Schmid et al., 2004; Vousden and Ryan, 2009; Contractor and Harris, 2012). Furthermore, several of the enzymes involved in glycolysis also “moon-light” as anti-apoptotic proteins or activate transcription, like glyceraldehyde 3-phosphate dehydrogenase (GAPDH) or hexokinase (Kim and Dang, 2005). Hexokinase 2, which phosphorylates glucose to glucose-6-phosphate, initiating the glycolytic pathway, is up-regulated in cancer and translocates to the mitochondria, where it inhibits the mitochondrial transition pore (MTP), suppressing apoptosis (Pastorino and Hoek, 2003; Kim and Dang, 2005). Hexokinase expression is increased by many oncogenic factors including HIF and AKT as well as loss of function mutations to p53 (Cross et al., 1995; Mathupala et al., 1997, 2001; Gottlob et al., 2001; Elstrom et al., 2004; Osaki et al., 2004). In other words, although in some tumors the initial hypoxic microenvironment in carcinogenesis may inhibit GO, this inhibition may persist by the collective action of many oncogenes. As will be apparent later on, the suppressed mitochondria can transmit “pseudo-hypoxic” signals that then further promote HIF activation, completing a powerful feed-forward feedback loop that sustains the glycolytic profile and the apoptosis resistance in the tumors.

The up-regulation of glycolysis follows the suppression of mitochondrial function and as discussed can promote tumor growth. There is also evidence that the inhibition of GO can also promote tumor growth, over and above the resulting up-regulation of glycolysis. For example, prevention of oxidation of pyruvate in the mitochondria and its metabolism in the cytoplasm has many consequences: **First,** by limiting the entry of pyruvate into the mitochondria, cancer mitochondria are deprived of their “fuel,” thereby decreasing their activity and directly preventing mitochondrial-dependent apoptosis. This can occur because apoptosis is initiated by the efflux of pro-apoptotic mediators from the mitochondria through the MTP, a mega-channel that is voltage- and redox-gated. The suppressed mitochondrial function in cancer cells promotes mitochondrial hyperpolarization and a decrease in the production of mitochondria-derived reactive oxygen species (mROS), both of which increase the opening threshold of the MTP, suppressing apoptosis (Chen, 1988; Bernardi, 1996; Zamzami and Kroemer, 2001; Hernandez-Garcia et al., 2010). **Second**, by metabolizing pyruvate in the cytoplasm by lactate dehydrogenase (LDH), cancer cells produce lactate and the resulting acidosis can facilitate the breakdown of the extra-cellular matrix, facilitating tumor expansion and metastasis (Gatenby and Gillies, 2004). **Third**, the prevention of pyruvate oxidation in the mitochondria, allows its use in anabolic biosynthetic pathways that are critical for the synthesis of several building blocks of rapidly proliferating cells (Deberardinis et al., 2008; Vander Heiden et al., 2009).

The GO inhibition comes at the expense of efficient energy production. GO provides ~36 adenosine triphosphate (ATP) per glucose molecule, compared to two ATP per glucose molecule produced during glycolysis. Therefore by increasing glycolysis, cancer cells may be able to generate a sufficient amount of ATP. To do this, cancer cells increase the expression of glucose transporters and glycolytic enzymes, to increase the uptake of glucose and enhance the glycolytic production of ATP. This explains why the PET imaging, which can measure glucose uptake in the tumor, remains perhaps the most sensitive tool to diagnose cancer. The therapeutic exploration of this metabolic remodeling has been significantly limited by the assumption that the glycolytic profile is a result of cancer progression and not a cause or a facilitator of cancer progression.

In summary, this newly discovered role of metabolism in both carcinogenesis and cancer progression, may be an essential requirement for tumor progression. Thus targeting the metabolic profile of cancer may be the basis for the development of effective and selective cancer therapies. The identification of therapeutic targets in cancer metabolism will become apparent by a more detailed review of the mechanisms that regulate apoptosis and pro-proliferative mitochondrial signaling.

## MITOCHONDRIA AND THE REGULATION OF APOPTOSIS AND PRO-PROLIFERATIVE REDOX SIGNALING

The intrinsic apoptotic pathway in cells is regulated largely by functional mitochondria (Green and Kroemer, 2004). Suppressing mitochondrial activity may give cancer cells a proliferative advantage to non-cancerous cells by suppressing apoptosis. How does the threshold for apoptosis relate to mitochondrial function?

The mitochondria regulate energy production through the oxidation of both pyruvate (derived from glycolysis) and lipids. GO begins with the entry of pyruvate into the mitochondria, where it is decarboxylated to acetyl-CoA by PDH. Acetyl-CoA is also produced by fatty acid metabolism in mitochondria. Acetyl-CoA then becomes the primary substrate for the Krebs’ cycle, from which the electron donors nicotinamide adenine dinucleotide (NADH) and flavin adenine dinucleotide (FADH_2_) are produced. Electrons are “donated” to complexes I and II of the electron transport chain (ETC), where they flow down a redox-gradient from complex I to complex IV, with molecular oxygen being the final electron acceptor (i.e., respiration). During this process, H^+^ are pumped from complexes I, III, and IV out of the inner mitochondrial membrane creating the mitochondrial membrane potential (∆ψm), which is quite negative (>200 mV). ATP synthase then uses the stored energy of the mitochondrial ∆ψm to phosphorylate adenosine diphosphate (ADP), producing ATP. Thus, since respiration is coupled to oxidative phosphorylation, ∆ψm may be an index of mitochondrial activity (Duchen, 1999).

Regulation of the mitochondrial ∆ψm is complex and depends on many factors. For example, mitochondria are critical regulators of intracellular calcium levels since they function as calcium “sinks,” particularly to the calcium secreted by the endoplasmic reticulum (ER). As the levels of the positively charged mitochondrial calcium rise, the ∆ψm decreases. Structural remodeling, altering the contact points between the ER and the mitochondria, as in ER stress, can also result in changes in the ∆ψm (Simmen et al., 2005; de Brito and Scorrano, 2008; Sutendra et al., 2011). Uncoupling proteins like uncoupling protein (UCP)1 or UCP2–3 (which conduct H^+^ and short-circuit the ∆ψm, or conduct calcium, respectively) can also regulate ∆ψm (Boss et al., 2000; Sack, 2006). In a glycolytic environment, inhibition of glycogen synthase kinase 3β (GSK3β) causes a translocation of hexokinase 2 onto the mitochondrial membrane, where it attaches to and inhibits the major pathway for the excretion of negative ions from the mitochondria, the voltage-dependent anion channel (VDAC; Pastorino et al., 2005); thus when the VDAC is inhibited the mitochondrial concentration of anions increases, increasing the ∆ψm. All of these parameters can contribute at variable degrees to the regulation of ∆ψm in response to metabolic signals or at different disease states. As ∆ψm increases, the opening of the MTP is decreased. MTP is a mega-channel, which includes VDAC and may be involved in mitochondrial membrane permeabilization (MMP) and the subsequent release of pro-apoptotic factors like cytochrome c and apoptosis inducing factor (AIF) from the mitochondria causing apoptosis (Crompton, 1999; Kroemer et al., 2007). The MTP is voltage-gated and thus the increase in ∆ψm increases its opening threshold suppressing apoptosis (Zamzami and Kroemer, 2001). In addition, mROS can also promote activation of the VDAC component of the MTP resulting in cytochrome c release (Madesh and Hajnoczky, 2001).

Thus ∆ψm and mROS are both regulated by mitochondrial function and particularly GO, and are directly involved in the regulation of apoptosis. Intriguingly, cancer mitochondria in the majority of cancers have hyperpolarized mitochondria compared to non-cancer cells. This was first described by Dr. Chen during his attempts to deliver drugs connected to positively charged molecules selective to cancer cells, taking advantage of the fact that cancer mitochondria were much more negative than non-cancer mitochondria (Chen, 1988).

We have shown that the increased ∆ψm, which we also found in several cancer cell types, was associated with a suppression of GO and suppression of mROS as well (Bonnet et al., 2007a; Michelakis et al., 2010; Sutendra et al., 2012). We showed that in contrast to what was believed at the time, this hyperpolarization and the suppression of GO were not a permanent result of mitochondrial dysfunction due to the cancerous process. When we increased GO, by “forcing” pyruvate’s entry into the mitochondria, increasing GO, the ∆ψm decreased, the mROS production increased, and the normal apoptotic thresholds were re-instituted in cancer cells, reversing the resistance to apoptosis (Bonnet et al., 2007a; Michelakis et al., 2010; Sutendra et al., 2012).

This normalization of mitochondrial function and GO had additional signaling effects, over and above the direct regulation of apoptosis through the opening of the MTP. For example, mROS can reach extra-mitochondrial redox-sensitive targets like transcription factors [HIF1α, p53, or nuclear factor of activated T cells (NFAT); Wang et al., 1995, 2000; Huang et al., 1996, 2000; Xie et al., 2001; Bonnet et al., 2007a] and Kv channels in the plasma membrane (Weir et al., 2005; Bonnet et al., 2007a), further facilitating apoptosis and decreasing the proliferative potential of cancer cells, as we discuss below.

In summary, there are multiple ways in which the metabolism of cancer cells (increased glycolysis and suppressed GO) is associated with mitochondrial remodeling (∆ψm hyperpolarization, decreased mROS production) that leads to resistance to apoptosis. A potentially simple way this metabolic profile and the suppression of apoptosis and redox signaling can be reversed is by the activation of the PDH, the gate-keeping regulator of GO. One particularly simple way is by the use of dichloroacetate (DCA).

## DICHLOROACETATE IN CANCER: PRE-CLINICAL WORK

**FIGURE 1:**
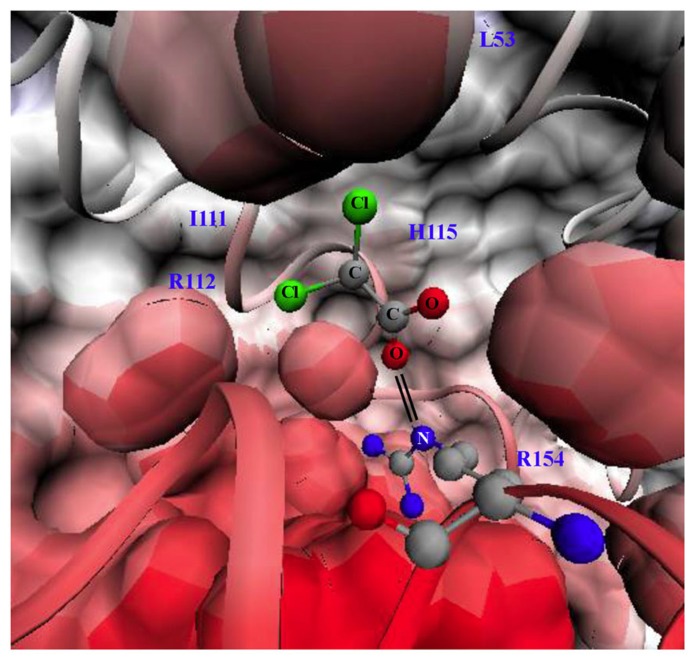
**Structure of pyruvate dehdyrogenase kinase 2 (PDK2) bound with dichloroacetate (DCA)**. Surface and ribbon representation of PDK2 with DCA bound is shown. The carboxylate group of DCA forms a salt bridge (double black line) with arginine 154 of PDK2. The structures were generated using visual molecular dynamics (VMD) version 1.8.6 with the coordinates acquired from Protein Data Bank (PDB#2BU8; Knoechel et al., 2006). L53 (leucine 53), I111 (lsoleucine 111), H115 (histidine 115), R112 (arginine 112), R154 (arginine 154). Black lines indicate salt bride between R154 and DCA.

Dichloroacetate is a small 150 Da molecule that can penetrate cell membranes and most tissues, even traditional chemotherapy sanctuary sites like the brain. DCA activates PDH by inhibiting PDK at a concentration of 10–250 μM, in a dose-dependent fashion (Stacpoole, 1989). There are four PDK isoforms that are expressed in most tissues with the most sensitive to DCA being PDK2. The crystal structure of PDK2 bound with DCA is shown in **Figure 1**: the carboxylate group of DCA forms a salt bridge with Arg-154 of PDK2 and the chlorine molecules reside in a hydrophobic pocket (Knoechel et al., 2006).

**FIGURE 2:**
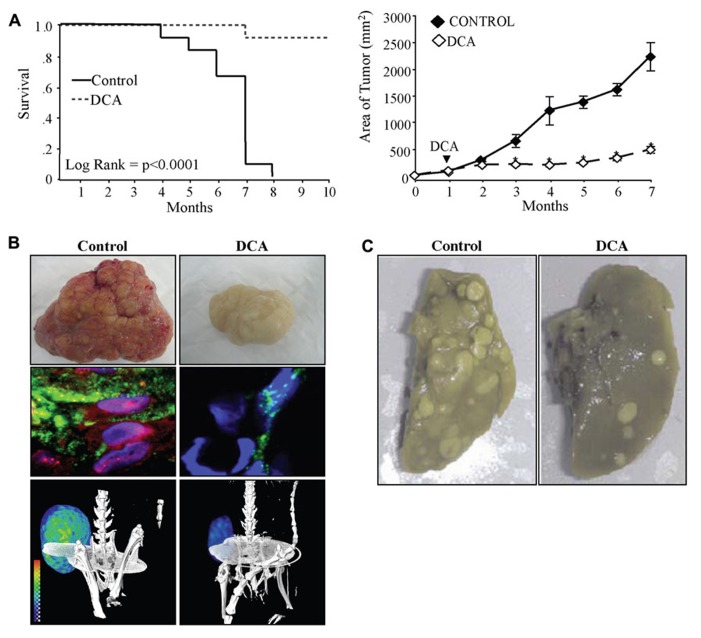
**(A)**DCA increases the survival (left) and decreases the tumor growth (right) of xenotransplant animals with nSCLC tumors. **(B)** DCA-treated animals have tumors with decreased vascularity (top) and decreased nuclear localization of hypoxia-inducible factor 1α (HIF1α; red, nuclear stain DAPI: blue) and angiogenesis (lectin: green, middle panel). DCA-treated animals have decreased glucose uptake in the tumors as indicated by PET imaging and ^18^-flurodexoglucose uptake (bottom panel). **(C)** DCA-treated animals have decreased metastases of the lung after tail vein injection of metastatic breast cancer. Figure modified with permission from (Sun et al., 2010; Sutendra et al., 2012).

Dichloracetate decreased (and normalized) the high ∆ψm in non-small cell lung cancer (nSCLC), breast cancer, and glioblastoma cancer cell lines, but had no effects on the normal low ∆ψm of non-cancer cells (Bonnet et al., 2007a; Michelakis et al., 2010; Sutendra et al., 2012). As expected, DCA promoted apoptosis of cancer cells *in vitro* and decreased tumor growth in xenotransplant models *in vivo *(Bonnet et al., 2007a; Sutendra et al., 2012). In these models, the mechanism of this small molecule was the specific inhibition of PDK2, as a PDK2 siRNA, completely mimicked DCA, and addition of DCA to the PDK2 siRNA had no additional effects (Bonnet et al., 2007a; Sutendra et al., 2012). The effects of DCA in cancer have now been extensively studied and the initial reports have been confirmed by several independent groups in other cancer types including colon (Sanchez-Arago et al., 2010), prostate (Cao et al., 2008), endometrial (Wong et al., 2008; Madhok et al., 2010), or metastatic breast cancer (Sun et al., 2010). In the latter study, the investigators showed that a number of breast cancer cell lines were sensitive to DCA, displaying decreased proliferation from 20 to 80% depending on the cell line. *In vivo*, DCA was effective in reversing aggressive metastatic breast cancer (Sun et al., 2010). The effects of DCA on tumor metastases in this study may be through inhibition of the redox-sensitive transcription factor HIF1α. In addition to increasing the expression of glycolytic enzymes, HIF1α can also increase the expression of pro-angiogenic and metastasis-promoting chemokines, such as VEGF and stromal-derived factor 1 (SDF1; Forsythe et al., 1996; Carmeliet et al., 1998; Pugh and Ratcliffe, 2003; Karshovska et al., 2007). HIF1α regulation is highly dependent on H_2_O_2_ and α-ketoglutarate (αKG; substrate of Krebs’ cycle; Huang et al., 1996; Wartenberg et al., 2003; MacKenzie et al., 2007), both of which are produced by mitochondria. For example αKG is a product of isocitrate dehydrogenase (IDH; a Krebs’ cycle enzyme) and can leak out of the mitochondria where it also serves as an important co-factor for the proline hydroxylases (PHD) that destabilize HIF (MacKenzie et al., 2007). Indeed, in other studies, DCA increased IDH activity and αKG levels in nSCLC, breast cancer, and glioblastoma cells (Michelakis et al., 2010; Sutendra et al., 2012). DCA decreased HIF1α activity resulting in decreased tumor angiogenesis, tumor perfusion, glucose uptake, and tumor size and increased animal survival (Sutendra et al., 2012 **Figures 2A,B**). These effects of DCA were confirmed in later studies showing that it decreases HIF1α activity in metastatic melanoma (Kluza et al., 2012) and T cell lymphoma (Kumar et al., 2012). This is in keeping with previous work showing that inhibition of PDK with short-hairpin RNA decreases HIF1α activity and tumor growth in squamous cell carcinoma (McFate et al., 2008). Therefore inhibition of PDK and activation of mitochondrial signals decreases HIF1α activity resulting in decreased tumor angiogenesis and tumor growth and may also potentially explain the decreased metastases observed in metastatic breast cancer (Sun et al., 2010; **Figure 2C**).

The suppression of mROS in cancer cells may promote a decrease in the activity of p53 in nSCLC and breast cancer cells and by increasing mROS production, DCA was also shown to increase p53 activity (Sutendra et al., 2012). The effect of DCA on p53 activity was also confirmed in T cell lymphoma (Kumar et al., 2012). The increase in p53 can sustain mitochondrial GO as p53 was recently shown to decrease the expression of PDK2 (Contractor and Harris, 2012). Activation of p53 can also result in decreased HIF1α activity since p53 can directly inhibit HIF1α transcriptional activity and promote HIF1α degradation (Kaluzova et al., 2004; Schmid et al., 2004; Sutendra et al., 2012).

In cancer cells the decrease in mitochondrial metabolism (i.e., decreased GO) would result in decreased ETC activity and decreased mROS (i.e., mostly superoxide) and ultimately decreased H_2_O_2_ production via the dismutation of superoxide (although the role of manganese superoxide dismutase (MnSOD) in cancer remains controversial and only partially explored; Holley et al., 2012). One would expect that the decreased activity of the ETC in cancer would also result in decreased pumping of H^+^ ions from the inner membrane, which would cause membrane depolarization, as less positive ions would leave the matrix. Yet, there is very strong evidence from the 1980s (Chen, 1988) and more recently (Pastorino and Hoek, 2003; Bonnet et al., 2007a; Michelakis et al., 2010; Sutendra et al., 2012) that cancer mitochondria are hyperpolarized, a fact that is in keeping with the apoptosis resistance characterizing these cells. There is no question that this important area needs more research, as clarification would be important for the emerging approaches to therapeutically target mitochondria or use them as the basis for diagnostic/imaging techniques. Here are some thoughts that we believe may help explain this apparent conflict: first, while less H^+^ are pumped out, less H^+^ also accumulates in the matrix as less electron and H^+^ donors (NADH, FADH_2_) are produced, following a potential decrease in pyruvate’s entry in mitochondria. Second, the decrease in mROS may also promote a closure of the redox-sensitive VDAC, i.e., the major anion channel in the mitochondria (Madesh and Hajnoczky, 2001). In addition, the resultant concomitant up-regulation of glycolysis is associated with a translocation of hexokinase 2 from the cytoplasm to the mitochondrial membranes, where it has been shown to bind and further inhibit VDAC (Pastorino and Hoek, 2003; Pastorino et al., 2005). In other words, less anions will also be leaving the mitochondria, contributing to an increase in ∆ψm. Furthermore, the accumulation of glycolysis-produced ATP in cytoplasmic microdomains associated with the mitochondria could serve as a signal to inhibit the ATP synthase (this intriguing possibility is difficult to be proven at this point given the absence of organelle-specific ATP imaging technologies). An increase in cytoplasmic ATP (and decrease in ADP) inhibits the gradient for ATP efflux from the mitochondria and thus decreases the drive for H^+^ re-entry into the matrix. Lastly more work in needed to clarify any potential role of mitochondrial uncoupling via uncoupling proteins, which short-circuit the H^+^ conduction through the mitochondrial membrane (Robbins and Zhao, 2011).

**FIGURE 3:**
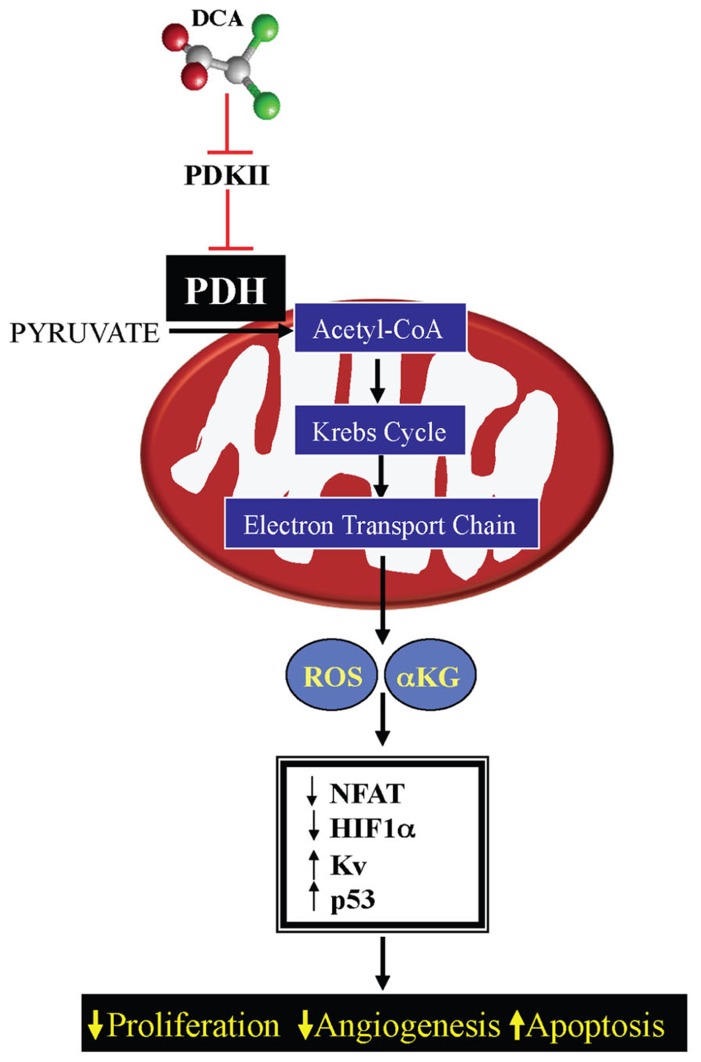
**Mechanism of DCA in cancer: DCA inhibits pyruvate dehydrogenase kinase (PDK) resulting in activation of pyruvate dehydrogensae (PDH)**. By increasing mitochondrial-based GO and acetyl-CoA levels, DCA increases Krebs’ cycle and electron transport chain activity, increasing mitochondrial-derived reactive oxygen species (mROS) and α-ketoglutarate (αKG) levels. This results in decreased activation of pro-proliferative transcription factors, like NFAT and HIF1α and increasing the activity of Kv channels and the tumor suppressor protein p53.

This decrease in mROS in cancer, in addition to the effects on HIF1α and p53, also results in inhibition of redox-sensitive K^+^ channels in the plasma membrane, specifically Kv1.5 (Bonnet et al., 2007a). The resulting decrease in K^+^ conductance causes an increase in the intracellular K^+^, which contributes to the suppression of apoptosis since K^+^ is a tonic inhibitor of caspases (Remillard and Yuan, 2004). The inhibition of Kv1.5 also results in depolarization of the plasma membrane and activation of L-type calcium channels, increasing intracellular calcium. Calcium can activate many enzymes involved in proliferation and mitochondrial remodeling, such as NFAT; Macian, 2005. NFAT is a transcription factor that can regulate the expression of many genes, increasing the expression of the anti-apoptotic protein B cell lymphoma-2 (bcl-2) and decreasing the expression of Kv1.5, thereby sustaining an apoptosis-resistant state (Bonnet et al., 2007b). All of these features may be secondary to mitochondrial remodeling. As discussed above, the initial switch toward glycolysis will also activate additional mechanisms (like the hexokinase translocation to the mitochondria), which also sustains mitochondria hyperpolarization and MTP inhibition. Therefore suppression of mitochondrial GO and the downstream mitochondrial signaling results in activation of HIF1α and NFAT and inhibition of p53, promoting proliferation, angiogenesis, and metastases, while the increased ∆ψm promotes MTP inhibition and apoptosis resistance. All of these effects are reversed by activation of PDH by DCA (Bonnet et al., 2007a; Michelakis et al., 2010; Sutendra et al., 2012; **Figure 3**).

Interestingly, in a recent study, two molecules of DCA were linked to cisplatin, a compound that induces nuclear cross-linking of DNA, resulting in apoptosis (Fuertes et al., 2003). This new molecule, mitaplatin, was selective to cancer cells inducing mitochondria-dependent apoptosis (through the effects of DCA), and nuclear degradation (through the effects of cisplatin), further validating the beneficial effects of DCA in cancer and providing evidence that DCA may have additional beneficial effects in combination with current anti-cancer therapies (Dhar and Lippard, 2009).

## CLINICAL EXPERIENCE WITH PDK INHIBITION BY DCA

### DCA IN TREATMENT OF CONGENITAL MITOCHONDRIAL DISEASES

In the past 40 years, DCA has been used extensively for the treatment of mitochondrial diseases. As first described in 1969 by Stacpoole (Stacpoole, 1969), by inhibiting PDK and coupling glycolysis to GO, DCA could lower lactate levels and the symptoms associated with lactic acidosis in patients with mitochondrial diseases, mostly syndromes caused by the lack or decreased expression of critical enzymes involved in GO, like PDH. As a result of the inhibited GO these patients suffer from lactic acidosis since pyruvate cannot be oxidized in the mitochondria and is metabolized to lactic acid by lactic dehydrogenase. In addition lactic acidosis complicates several non-mitochondrial diseases in which tissue hypoxia and hypo-perfusion results to metabolic acidosis, including sepsis or heart failure. Thus, DCA has been tested in clinical trials in both mitochondria and non-mitochondrial diseases in order to limit lactic acidosis (Stacpoole et al., 2003). As expected, while DCA was effective in decreasing lactic acid levels, it did not improve the outcomes of these patients, as it can only limit a symptom of these diseases and not their primary cause (lack of a critical enzyme or the original cause for decreased tissue perfusion etc). In the first study 30 patients with MELAS syndrome were treated with 25 mg/kg/day of oral DCA. Most of these patients had developed symptomatic peripheral neuropathy, compared to the placebo group. This resulted in early termination of the study. Seventeen out of the 19 patients who had reported peripheral neuropathy had resolution of the neuropathy following 9 months discontinuation of DCA. No other toxicities were reported in that study (Kaufmann et al., 2006). Overall, this non-demyelinating peripheral neuropathy is known to be reversible and dose-dependent. In a second placebo-controlled double-blinded study, oral DCA treatment (25 mg/kg/day) of 21 children with congenital lactic acidosis demonstrated mild peripheral neuropathy at 6 months. However, serial nerve conduction studies showed no detectable differences in incidence of neuropathy between placebo and DCA-treated groups (Stacpoole et al., 2006). This would indicate that the higher incidence of peripheral neuropathy in the MELAS group might be due, in part, to the natural history of the disease, as patients with MELAS also have diabetes or diabetes-related peripheral neuropathy.

### DCA IN GLIOBLASTOMA MULTIFORME

**FIGURE 4:**
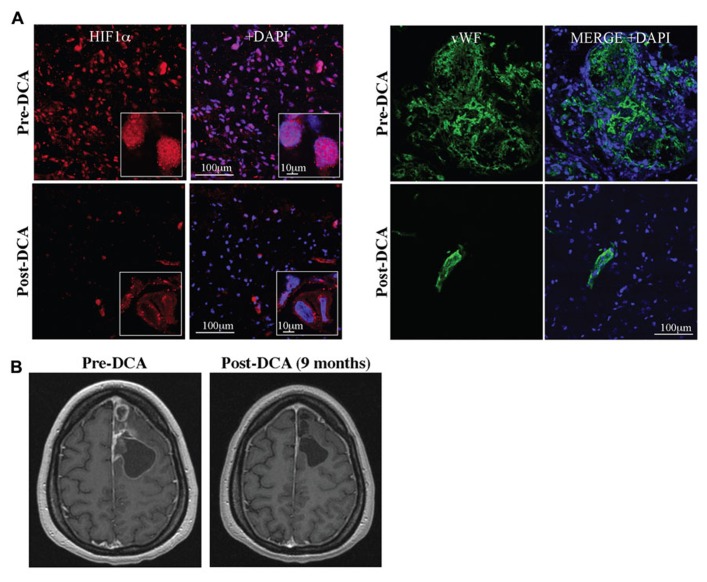
**(A)** DCA decreases nuclear localization of HIF1α (HIF1α, red; merged with DAPI, blue; right) and angiogenesis in GBM (vWF, green; merged with DAPI, blue; right). **(B)** DCA decreases tumor growth in a patient with GBM after 9 months of treatment. Figure modified with permission from (Michelakis et al., 2010).

The solid evidence in pre-clinical *in vitro* and *in vivo* models, suggested that DCA might be beneficial in human cancer. Glioblastomas are aggressive, highly vascularized tumors for which there are no cures and available treatments prolong life only minimally. The expected survival in newly diagnosed GBM is just over a year whereas the survival of recurrent disease (despite treatments) is less than 6 months (Wen and Kesari, 2008). We first studied 49 freshly excised GBM tumors and compared them to normal brain tissues obtained during epilepsy surgery, studying them immediately with functional confocal microscopy imaging. GBM tumors had increased ∆ψm compared to normal brain tissue (Michelakis et al., 2010). Similar to pre-clinical studies, DCA acutely depolarized mitochondria in these GBM tumor tissues, but had no effect on normal tissues (Michelakis et al., 2010). In addition, the target of DCA (i.e., PDK2) was expressed at much higher levels in the GBM compared to the normal brain tissues (Michelakis et al., 2010). We then administered DCA orally at a dose of 12.5 mg/kg twice a day in five patients with primary GBM (Michelakis et al., 2010). Three of these patients had recurrent GBM with disease progression after several chemotherapies. None of these patients developed hematologic, hepatic, renal, or cardiac toxicity. Peripheral neuropathy was noted, but reversed upon a dose-decrease to 6.25 mg/kg of DCA twice a day. At a dose of 6.25 mg/kg orally twice a day the trough levels of DCA after 3 months of therapy were comparable to those in DCA treatment of adults with mitochondrial defects and in the range of the K_i_ of DCA for PDK2 inhibition (0.2 mM; Bowker-Kinley et al., 1998). Three of the patients showed evidence of radiologic regression, while four of the five patients were clinically stable at 18 months of DCA therapy (Michelakis et al., 2010). GBM tissues from three of these patients were obtained prospectively (pre-DCA) and compared with the tissues after DCA treatment (post-DCA) from the same patients. In post-DCA GBM tissues, there was a significant decrease in ∆ψm and increase in mROS. Chronic treatment of DCA resulted in decreased proliferation and increased apoptosis *in vivo* compared to the same patients pre-DCA GBM biopsies. As expected, by inhibiting PDK, DCA increased PDH activity in the post-DCA GBM tissues compared to the pre-DCA GBM tissues. It also appears that DCA may have effects on putative GBM stem cells (GBMSC), which are responsible for tumor recurrence. GBMSC had significantly increased mitochondrial ∆ψm compared to the primary GBM tumors, both *in situ* in freshly excised tumor tissues and *in vitro* (GBMSC were isolated using magnetic beads from the excised tumors and allowed to form neurospheres), compatible with the fact that these GBMSC are even more apoptosis resistant than primary tumor cells. DCA depolarized mitochondria, increased mROS, decreased proliferation, and induced apoptosis in GBMSCs, similar to its effects on the primary GBM cells. Additionally, post-DCA GBM tumor biopsies had decreased HIF1α expression and activity resulting in a significant decrease in tumor vascularity compared to pre-DCA GBM biopsies (**Figure 4**). This was expected as DCA increased both H_2_O_2_ and αKG, molecules that can increase HIF1α inhibition (Huang et al., 1996; MacKenzie et al., 2007). Mutations in the genes for IDH (an enzyme that catalyzes the decarboxylation of isocitrate to αKG) have been described in GBM (Dang et al., 2009; Thompson, 2009) and these mutations could result in activation of HIF1α, in part by decreasing αKG levels (MacKenzie et al., 2007). In addition, DCA increased p53 levels and its downstream target, p21, which may also contribute to the decreased proliferation shown in the DCA-treated tissues *in vitro* and *in vivo *(Michelakis et al., 2010). With no significant toxicity, its relative selectivity and its potential beneficial effects on tumor proliferation, angiogenesis, and apoptosis, DCA holds promise in the emerging new frontier in metabolic oncology. This mechanistic study in patients with GBM, although small, established the fact that after oral therapy DCA can reach levels high enough to have the same effects in human tumors as in the pre-clinical studies, showing feasibility of this novel approach in humans.

### THE MECHANISM OF DCA MAY INVOLVE ADDITIONAL ASPECTS OF METABOLISM

While this is not reviewed here, the DCA results may also be compatible with exciting data suggesting that targeting fatty acid β-oxidation (FAO) may also be important as a potential cancer treatment (Samudio et al., 2010). In most cell types there appears to be balance in the regulation of the primary mitochondrial fuel for oxidative phosphorylation, i.e., pyruvate versus fatty acids. In fact, Randle (1998) first described an elegant pathway by which inhibition of FAO results in activation of PDH and thus promotion of GO, and vice versa (the Randle feedback). In this mechanism, Randle (1998) described how acetyl-CoA, NADH, and ATP produced by FAO could directly activate PDK, therefore decreasing GO. Thus, the inhibition of FAO indirectly promotes GO in a manner that mimics the direct activation of GO by DCA. Lastly, the suppression of GO in cancer allows pyruvate to also enter other important pathways, like amino-acid biosynthesis reactions that are critical for protein synthesis in rapidly proliferating cells and their independence of exogenous trophic factors (Quash et al., 2003). In other words, refueling pyruvate into the mitochondria with DCA, may also lead to disruption of these biosynthesis pathways that are activated in cancer cells, suggesting that additional mechanisms may play a role in the overall effects of DCA *in vivo*.

## OTHER METABOLIC THERAPIES THAT MIMIC PDK INHIBITION AND PROMOTE MITOCHONDRIAL GO

Although DCA can selectively increase mitochondrial metabolism in cancer cells, there are other ways to increase mitochondrial activity. LDH is an enzyme that completes pyruvate metabolism to lactate in the cytosol. When LDH is inhibited, pyruvate may be “forced” to enter the mitochondria and undergo decarboxylation to acetyl-CoA, increasing GO. Remarkably, similar to DCA, inhibition of LDH-A by short-hairpin RNAs increased mitochondrial activity, depolarized mitochondria, inducing apoptosis, and decreasing tumor growth *in vitro* and *in vivo, *once again mimicking DCA (Fantin et al., 2006).

In addition, recent data have shown that the M2 isoform of pyruvate kinase (PKM2) is highly expressed in many cancers compared to the M1 isoform of pyruvate kinase (PKM1), which is mostly expressed in non-cancerous tissues (Christofk et al., 2008; Vander Heiden et al., 2010). It is now known that this less active form of pyruvate kinase (PKM2), results in mitochondrial suppression and tumor growth (Christofk et al., 2008; Anastasiou et al., 2012). Replacement of PKM2 with PKM1 or increasing PKM2 activity both result in increased mitochondrial GO and decreased tumor growth (Christofk et al., 2008; Anastasiou et al., 2012). This suggests that similar to PDK, PKM2 may also be a viable and selective target against cancer.

It is now becoming increasingly recognized that targeting the metabolism of cancer cells may offer a selective and effective anti-cancer therapy. However, it is important to clarify that inhibiting glycolysis would not be selective to cancer cells or increase mitochondrial metabolism. In fact, it would be toxic to non-cancerous tissues that depend on glycolysis for energy production (i.e., skeletal muscle or brain tissue). In other words, drugs that inhibit glycolysis do not necessarily result in an increase in GO and could simply result in disruption of energy production and non-selective cell death, an undesired feature of promising cancer drugs. It is more important to promote coupling of glycolysis to GO (which in turn would eventually result in decreased glycolysis, by decreasing the activity of pro-glycolytic transcription factors, like HIF1α) as this is the strategy that would re-activate the suppressed mitochondria-dependent apoptosis just in cancer cells as opposed to inducing energy starvation and cell death in all tissues utilizing glycolysis for energy production.

## CONCLUSION

In summary, a diverse number of signaling pathways and oncogenes (i.e., loss of p53 function, activation of HIF1α, cmyc, etc.) result in resistance to apoptosis and a glycolytic phenotype. DCA can reverse this metabolic remodeling and may be an effective anti-cancer agent in a number of tumors. The pre-clinical and recent clinical evidence that DCA, as a single agent, might be effective in GBM (Bonnet et al., 2007a; Michelakis et al., 2010), suggests its use in other glycolytic forms of cancer. However, the greatest effect may be synergistic with existing chemotherapy as DCA “unlocks” cancer cells from a state of apoptosis resistance. Preliminary evidence in GBMSC, in which the highest induction of apoptosis was achieved with the combination of DCA and temozolomide, supports this idea (Michelakis et al., 2010). Therefore, in future clinical trials, DCA could be given simultaneously with standard therapy in an attempt to increase its effectiveness and potentially decrease the required dose, limiting the toxicity of standard therapies, which often are non-selective to non-cancerous tissues. The ability to target the unique metabolic profile of cancer cells, suggests that imaging and diagnostic studies, such as PET imaging, may track the metabolic modulation of therapies like DCA. Finally, pre-clinical biopsies from patients can acutely be treated with DCA and mitochondrial function can be quickly assessed, potentially predicting the clinical response to DCA, which could be used to facilitate patient selection.

## Conflict of Interest Statement

The authors declare that the research was conducted in the absence of any commercial or financial relationships that could be construed as a potential conflict of interest.
